# Stochasticity Versus Determinacy in Neurobiology: From Ion Channels to the Question of the “Free Will”

**DOI:** 10.3389/fnsys.2021.629436

**Published:** 2021-05-26

**Authors:** Hans Albert Braun

**Affiliations:** Neurodynamics Group, Institute of Physiology and Pathophysiology, Philipps University of Marburg, Marburg, Germany

**Keywords:** noise, randomness, consciousness, volitional decisions, readiness potentials, non-linear feedback, synchronization

## Abstract

If one accepts that decisions are made by the brain and that neuronal mechanisms obey deterministic physical laws, it is hard to deny what some brain researchers postulate, such as “We do not do what we want, but we want what we do” and “We should stop talking about freedom. Our actions are determined by physical laws.” This point of view has been substantially supported by spectacular neurophysiological experiments demonstrating action-related brain activity (readiness potentials, blood oxygen level–dependent signals) occurring up to several seconds before an individual becomes aware of his/her decision to perform the action. This report aims to counter the deterministic argument for the absence of free will by using experimental data, supplemented by computer simulations, to demonstrate that biological systems, specifically brain functions, are built on principle randomness, which is introduced already at the lowest level of neuronal information processing, the opening and closing of ion channels. Switching between open and closed states follows physiological laws but also makes use of randomness, which is apparently introduced by Brownian motion – principally unavoidable under all life-compatible conditions. Ion-channel stochasticity, manifested as noise, function is not smoothed out toward higher functional levels but can even be amplified by appropriate adjustment of the system’s non-linearities. Examples shall be given to illustrate how stochasticity can propagate from ion channels to single neuron action potentials to neuronal network dynamics to the interactions between different brain nuclei up to the control of autonomic functions. It is proposed that this intrinsic stochasticity helps to keep the brain in a flexible state to explore diverse alternatives as a prerequisite of free decision-making.

## Introduction

The question of whether humans have free will has been debated since antiquity. Nowadays, such attacks do not necessarily come from professional philosophers. Rather, it is renowned representatives of the neurosciences who have been repeatedly questioning the free will of humans for a number of years. Of course, it is not a surprise that neuroscientists contribute to this discussion. In fact, it is their tasks to investigate the functions of the nervous system, which also includes the question of the neuronal basis of higher mental and cognitive processes up to decision-making.

The possibility of free decisions could actually be regarded as an empirically well-proven fact if there were no assumptions that these everyday experiences of free will are nothing more than an illusion. The rationale is that decisions are made in the brain whose functions are subject to deterministic laws of nature. It is claimed that our decisions are determined before we even perceive them as our own will.

This assumption was strongly supported by neurophysiological data such as from the often quoted Libet experiments (Libet et al., 1982, 1983a) and more recent experiments from the Haynes group (Soon et al., 2008; Schultze-Kraft et al., 2016). These experiments essentially demonstrate that action-related brain signals, readiness potentials in the electroencephalogram (EEG), and blood oxygen level–dependent (BOLD) signals in functional magnetic resonance imaging (fMRI) data can be recorded before a test person becomes aware of his/her decision to perform the action. Such studies have been and still are controversially discussed concerning technical issues as well as the interpretation of the results, specifically with regard to the question of a free will (Heisenberg, 2009; Smith, 2011; Mele, 2014). As even the experimentalists apparently were not so happy about having destroyed the idea of a free will, there were diverse ideas and experimental attempts to save the free will, e.g., with the help of “deterministic chaos” or by invention of a “veto right,” partly even experimentally (Libet et al., 1999; Libet, 2005; Searle, 2007; Schultze-Kraft et al., 2016; Maoz et al., 2019b; Travers et al., 2019). This article will essentially focus on one specific question, which typically comes up in these discussions, namely, to what extent a free decision can be possible in an otherwise eventually completely deterministic world.

Indeed, determinacy is a really fundamental issue that can stand for itself to question the existence of a “free will,” independent of the aforementioned neurophysiological experiments. However, statements as even heard from neuroscientist saying “there are no indications of indeterminacy in the brain” would immediately be objected to by all experimental neurophysiologists. It is their everyday experience to deal with an enormous diversity of neuronal activity in the brain. Experimentalists always have to struggle with the fact that no neuron reacts in the same way as any other one and that even the same neuron always reacts differently on repeated application of identical stimuli under identical experimental conditions.

The main question is whether this variety is simply due to the lack of our knowledge in view of the complexity of multiple meshed brain functions or whether it eventually reflects a principle uncertainty, which becomes specifically pronounced in biological systems. This study proposes that biology, indeed, has found a way to escape from full determinacy due to the organizational principles on which biological systems are based. Even more, it can be shown that these systems apparently have learned harnessing stochasticity, taking advantage of the molecular randomness in thermodynamics. It will be demonstrated by experimental recordings, supplemented by computer simulations, that biological systems, specifically brain functions, are built up on principle randomness, far above eventual quantum uncertainty but already manifested at the lowest level of neuronal information processing: the opening and closing of ion channels. Ion-channel transitions, indeed, follow physiological laws but apparently also need to make use of randomness presumably originating from Brownian motion which is principally unavoidable under all life-compatible temperatures.

Moreover, it can be demonstrated that this randomness will not necessarily smear out toward higher functional levels of action potential (AP) generation and neuronal network activity but can even be amplified by cooperative effects with the system’s non-linearities. In this way, harnessing stochasticity can help to keep the brain in a state of flexible and principally unpredictable information processing, thereby offering the choice between different options as a basis for free decisions, irrespective of whether or not they later turn out to be more or less appropriate.

The article will conclude with a discussion about specific features of biological functions to harness stochasticity, also in comparison with the purely physical world and conventional engineering. Possible implications concerning the free will shall be discussed with specific regard to the question of determinacy or stochasticity.

## Results—Harnessing Stochasticity

Stochasticity or randomness is omnipresent in nature and seems to be even more pronounced in biology than in physics. Is this due to the particular complexity of living systems, or does it reflect an additional randomness introduced with the emergence of life? The random processes known from physics, such as thermodynamics or an indeterminacy brought about by quantum mechanics, also have effects in inanimate nature.

In the following it is proposed that differences between inanimate and animate world are not necessarily to be found in the nature of the random processes, but rather in the specificity of biologically organizational principles – and above all in how they “exploit” chance. The latter is particularly evident at the cellular level in controlling one of the fundamental life processes: the opening and closing of ion channels. This obviously happens by using thermodynamic random processes, far above quantum physics and seemingly without any need to bring coincidence into play.

This interplay of coincidence and lawfulness is a very effective way to control system behavior, at least biologically – not with the precision of a computer, but perhaps with greater flexibility. The principle is based on bringing the system close to a so-called bifurcation, i.e., to a kind of “threshold,” where smallest random fluctuations decide whether it tilts in one direction or another.

Such situations can be found at all levels of biological systems, from ion channels to cognitive processes. The fact that the random processes brought in at the lowest level are not necessarily smoothed out toward higher levels, but rather intensified, is due to the close connection of the different levels via multiple networked, non-linear and time-delayed, positive and negative feedback circuits, from which new bifurcations with threshold behavior result again and again.

Coincidence is an integral part of everyday life. Everyone has heard or proclaimed the phrase “Such a coincidence!” This usually happens in response to a surprisingly unexpected event. However, no one is likely to come up with the idea of associating such coincidences with the uncertainties of physics. As far as everyday life is concerned, the laws of nature are regarded as quite stable.

However, the term “coincidence” is not only used in colloquial language, but also in the language of science. In fact, not only everyday life seems to be full of coincidences. Publications in the life sciences are full of data on mean values, standard deviations, and other statistical parameters. Such measures can, of course, also be found in the so-called exact natural sciences. But in animated nature, the variability seems to be particularly pronounced. Of course, it is difficult to prove that such variability is not only due to the manifold of unknown influences that cannot be kept under control also in perfectly designed experiments. There are, however, specific, well-known experiments in which random effects can directly be observed essentially contributing to the system’s function. This is only possible in most simple and basic systems in which it can be assumed that they are not yet subject to a large number of undetectable influencing factors. Accordingly, the most concrete and experimentally well-proven indications of functionally relevant random effects were obtained in recordings of single-ion-channel openings and closing of isolated cells, most convincingly in the excised patch technique (Neher and Sakmann, 1976, 1992).

### Random Processes for Controlling Ion Channels

**FIGURE 1:**
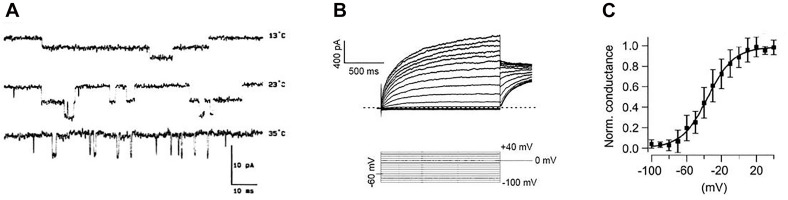
Examples of experimental “patch-clamp” registrations of ion currents. **(A)** Single-channel currents (downward swings) through acetylcholine receptors at different temperatures [from Dilger et al. (1991) with permission by “Elsevier”]. **(B)** Voltage-dependent whole cell currents (top) in response to voltage steps to different membrane potentials (bottom). **(C)** Curve of the voltage-dependent activation of ion channels plotted as normalized conductivity (measured current related to maximum current). Mean values of repeated measurements are plotted together with the standard deviations and fitted to a Boltzmann function (solid line) [Figures 2B,C from Leitner et al. (2012) with permission by “John Wiley and Sons”].

Recordings of single-ion-channel openings and closings provide concrete and experimentally well-proven indications that biology harnesses stochasticity very effectively, already at the lowest level of neuronal functions. These processes, indeed, are subject to physiological laws. However, these laws only determine the probability with which the channel states change, e.g., as a function of the membrane potential or in dependence of certain molecules such as neurotransmitters (as in Figure 1A) or hormones. The switchover itself is purely random. No regularity could be found. Also, all attempts to relate the transitions to deterministic chaos were without success. The transitions between open and closed are determined by stochastic processes (Hille, 1978; Kandel et al., 1991; White et al., 1998, 2000; Alvarez et al., 2002).

In search for the source of this randomness, one does not need to go down to the uncertainty of quantum mechanics. The pronounced temperature dependencies as seen in Figure 1A indicate that this kind of coincidence is likely introduced by thermal noise, i.e., by Brownian motion. Therefore, it is perhaps no coincidence that the opening probabilities of the entire ion channels, determined experimentally from the current–voltage curves, are preferably fitted to the Boltzmann function. The experimental data in Figure 1 show single-channel currents (Figure 1A) together with measurements of the total current of a cell (Figure 1B) and the Boltzmann function fitted to the thereby obtained values (Figure 1C).

**FIGURE 2:**
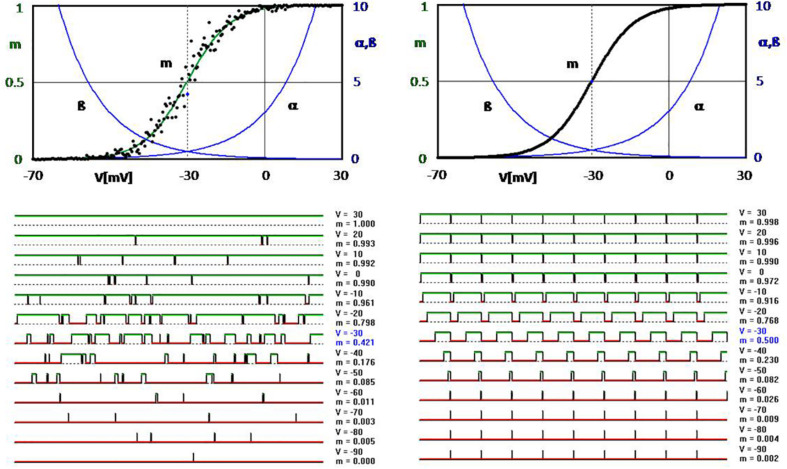
Computer simulation of voltage-dependent opening and closing of ion channels (simplified approach according to Hodgkin and Huxley, 1952; see Tchaptchet et al., 2013). **Upper diagrams:** Exponential transition functions (rate constants) α and β with *p* = α/(α + β) leading to the well-known Boltzmann function of the opening probability (all shown in green). The black dots scattering around it in the upper left-hand diagram are obtained when the transitions are determined by drawing random numbers in comparison to the values given by α and β. Some examples of this are shown in the graphs at the bottom left (over a 60-ms time axis with a calculation step size of 0.1 ms). The curves at the bottom right are obtained by determining the opening and closing times directly from the transition functions α and β without random factors. Accordingly, all points of the opening probability lie exactly on the Boltzmann curve in the upper right diagram, which then is completely covered by the black dots.

It is noteworthy that these basic cellular processes controlling the state of ion channels are harnessing stochasticity seemingly without need. To illustrate this, computer simulations of voltage-dependent ion-channel openings and closings are shown in Figure 2, which were modeled according to the principles first described by Hodgkin and Huxley in the middle of the last century (Hodgkin and Huxley, 1952) and frequently confirmed. For the left figure, the transitions were determined by random computer values compared with the respective values of the voltage-dependent transition rates α and β (Tchaptchet et al., 2013). In this way, one obtains the typical time course of random opening and closing, as it is also observed experimentally (Figure 2, bottom left). Accordingly, the values of the open times (dots in the upper graph of Figure 2), calculated from such simulations, scatter randomly around the deterministically expected Boltzmann function (green) as calculated from the exponential functions (blue).

However, in principle, it would not be any problem at all to open and close without any random numbers, but by exactly determined switching intervals calculated from the relation between open and closed states according to the transition variables. This is shown in the figure on the right. However, such an image has never been observed experimentally – not even as an approximation. Even the opening probabilities when measured over a longer period of time will never, or only by chance, hit exactly the deterministic curve. The measured values are always scattered as in the upper left-hand corner of the simulation picture, corresponding to the standard deviations of the experimental registrations in Figure 1C. It would take an infinite time to obtain an exact reproducible value.

At first glance, it does not seem to be a good choice to use stochasticity to control such a fundamental process. If an engineer had to construct a gradually voltage-dependent function, then he would hardly come up with the idea of installing a random component. It could cost him his job. Probably the engineer’s curve would not even have a sigmoid form but would be linear, with threshold and maximum value. The Boltzmann function itself reminds one too much of coincidence.

That the system also works with coincidence is only because nature has obviously adjusted the ion channels in such a way that even the slightest random fluctuations, e.g., due to Brownian motion, can determine the opening and closing of the channels. In this situation, minimal shifts of the channels’ operating range by physiological control parameters (membrane potential, neurotransmitters, hormones, etc.) can be significantly amplified to increase or decrease the random effects in the one or the other direction. This is how cooperative effects between physiological laws and stochasticity can be used to adjust the opening and closing probabilities of the ion channels – and thus the strength of the ion currents.

Of course, this will never lead to exactly reproducible values as in the simulation of Figure 2 on the right. However, how should such an exactly determined curve be reproduced by an ion channel that can only switch between an open or closed state? The cell is not a computer that can be used to convert the voltage dependencies into time intervals. It cannot calculate like a digital computer, and it also does not have a random-number generator. It has to make use of what is available, e.g., the randomness of Brownian motion. This secures stochasticity an outstanding place in the animated world already at the lowest level of cellular processes.

In this sense, one may regard it as an ingenious invention of nature to adjust the ion channels transition states in such a way that it only needs to make use of the, in any case, ubiquitous thermodynamic noise to switch the ion channels according to their physiological laws. And this principle seems to run through all levels of physiological processes. Whenever so-called “bifurcations” occur, smallest random effects can determine whether the system switches in the one or in the other direction. This also applies to the next higher level, where APs are triggered.

### Randomness as “Noise” When Triggering Action Potentials

The opening and closing of ion channels determine the electrical activity of a cell, but the communication between nerve cells takes place largely via APs, The triggering of APs requires a certain depolarization of the cell up to a certain so-called “trigger threshold,” at which the Na channels open like an avalanche. If the membrane potential of a cell comes close to this “threshold,” even the smallest random fluctuations, as background noise so to speak, can decide whether or not an AP is triggered.

**FIGURE 3:**
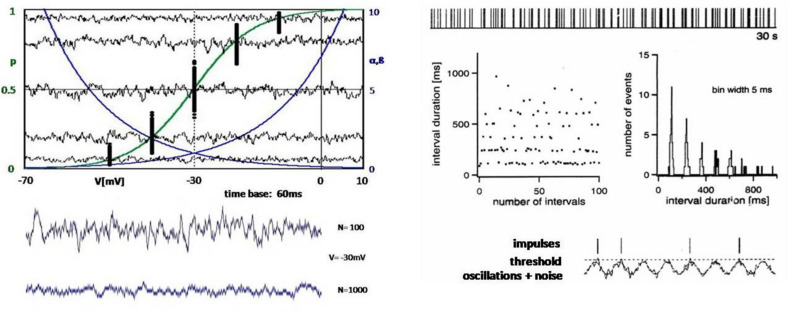
**Left:** Computer simulations of the opening states of ion channels with randomly introduced variability (according to the left-hand diagrams in Figure 2), which manifests itself as noise over time. The upper diagram again shows the exponential transition functions α and β (blue) and the resulting Boltzmann function p (green). The black dots are the opening probabilities averaged over 60 ms according to the points in Figure 3 (top left), but are now determined for repeated passes with a few potentials. In addition, the fluctuations of the opening probabilities are shown as a sum of 100 ion channels over the period of 60 ms. The lower diagram shows another curve of the fluctuations at –30 mV with 100 ion channels and at a 10-fold increase to 1,000 channels. **Right:** Pulse sequence, interspike intervals, and interval histogram to illustrate a neuronal pulse pattern whose structure implies that the action potentials are generated by intrinsic oscillations with functionally decisive participation of random processes (noise) (Braun et al., 1994).

Noise is the dynamic aspect of stochasticity, as it manifests itself in the form of fluctuations along the time axis. On the left in Figure 3, instead of the individual channels shown in Figure 2, random current fluctuations are plotted as the sum of many ion channels. The amplitude of the fluctuation, or the noise intensity, changes according to the slope of the curve of the potential-dependent opening probability (Figure 3, top left). Of course, the fluctuations also become smaller with increasing number of ion channels (Figure 3, bottom left). But it would take an infinite number of ion channels to actually eliminate the noise.

A particularly convincing experimental example of the physiological significance of noise was found in the analysis of AP sequences recorded from thermosensitive electroreceptors of sharks, the so-called ampullae of Lorenzini (Braun et al., 1994). These receptors belong to probably the most susceptible sensors, again with particular help of chance. In this case, it is an oscillating membrane potential that obviously comes so close to the trigger threshold that small random fluctuations can decide whether the threshold is exceeded, i.e., whether an AP is triggered or not (Figure 3, bottom right). This leads to characteristic impulse patterns with interspike intervals distributed around integer multiples of a basic period of impulse generation (Figure 3, right, upper diagrams).

It is immediately obvious that the slightest changes of the oscillation amplitude or minimal shifts of the oscillation baseline by any kind of stimulus can dramatically alter the probability of AP generation and thus making smallest signals perceptible. It is noteworthy that these sensory systems keep coming back to this highly sensitive subthreshold regime of stimulus encoding under all conditions, even after severe disturbances.

These sensory cells are vital for the sharks’ navigation and prey detection. Hence, the shark, as an evolutionary very old species, would eventually have hardly survived for so long a time, over millions of years, without the capability of harnessing noise. The same principle of AP generation based on oscillations and noise is used in many other cells in the peripheral and central nervous system (CNS) (Braun et al., 2003; McDonnell and Ward, 2011) – albeit nowhere else as excessively and exclusively as in electroreceptors of sharks.

### Synapses and the Stochasticity of Voltage- and Ligand-Controlled Ion Channels

At higher functional levels, it is naturally becoming increasingly difficult to distinguish a true random process from the opaqueness of an increasing number of possible influencing factors. This also applies to the triggering of APs in neurons in the CNS, which are usually subject to a wide variety of synaptic influences from many other nerve cells. Often, synaptic transmission, especially the release of a large, unmanageable number of transmitter molecules, is seen as the main source of neuronal randomness (White et al., 1998; Heisenberg, 2009). This is probably correct but is difficult to prove because of the manifold.

Anyway, to demonstrate stochasticity also at the synaptic level, it is sufficient to remember that also synaptic transmission, at different steps, involves opening and closing of diverse types of ion channels (Postnova et al., 2010). First, there are the voltage-gated Na^+^ and K^+^ channels carrying the incoming, presynaptic AP. The next step is the opening of voltage-dependent Ca^++^ channels at the presynaptic ending. This initiates then a whole sequence of biochemical processes that finally lead to transmitter release. The transmitters, in turn, interfere with ion channels at the postsynaptic membrane, thereby directly or indirectly opening so-called ligand-gated ion channels at inotropic or metabotropic receptors. The thereby induced potential changes, again, lead to the opening or closing of voltage-gated channels at the postsynaptic membrane, eventually triggering a postsynaptic AP. Incidentally, the registrations in Figure 1, left, show single-channel currents through ligand-gated ion channels.

**FIGURE 4:**
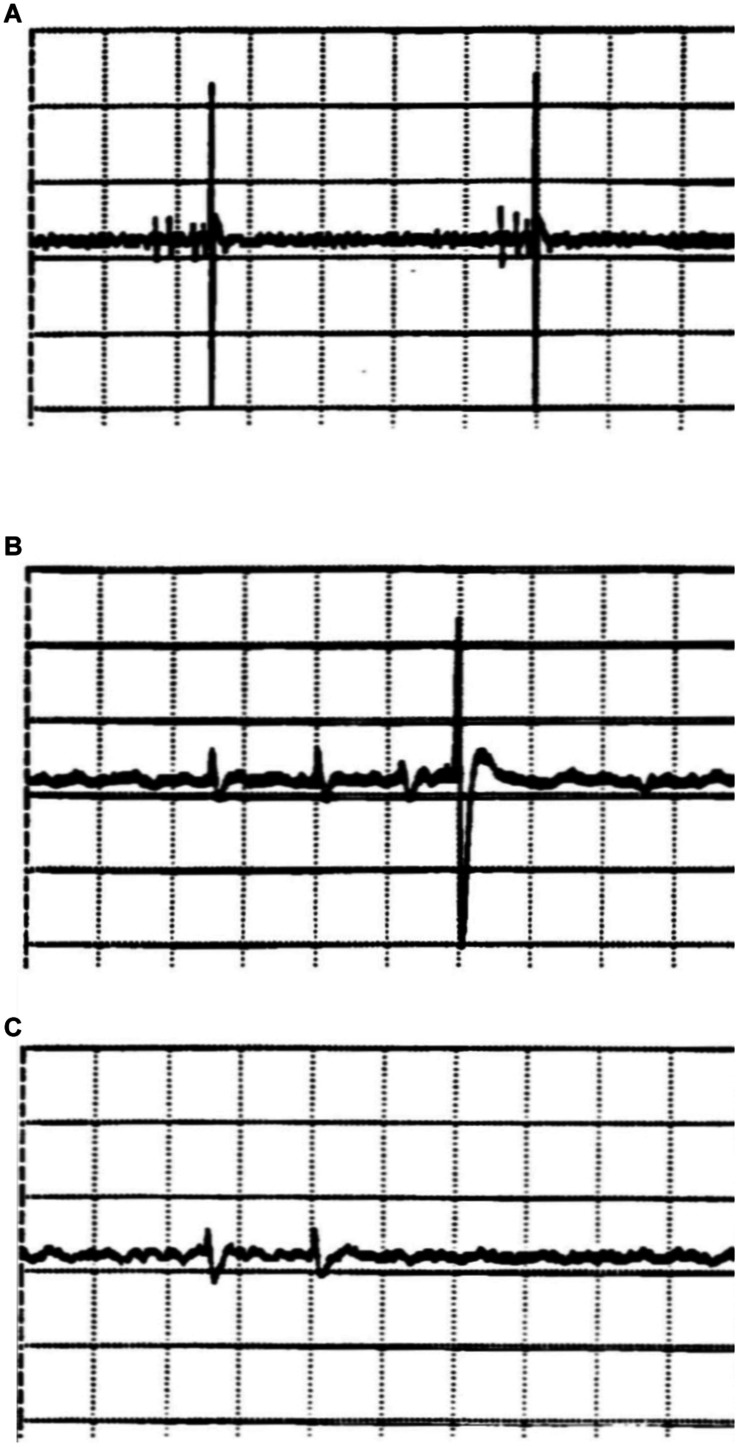
Extracellular registrations of action potentials from hypothalamic brain slices of the rat (nucleus paraventricularis). The figures, screenshots from the oscillograph, show the action potentials (APs) of two different neurons, which can be distinguished by their very different amplitudes (ordinate: 50 μV/Div). Groups of small action potentials are followed by a single large action potential (**A**, time base: 200 ms/Div). The lower traces, plotted in higher time resolution (50 ms/Div), show a burst triplet of small spikes followed by a big spike **(B)**, whereas a big spike does not occur after a burst doublet **(C)**.

An experimental registration of APs from hypothalamic brain slice preparations of the rat illustrates how randomness in synaptic transmission may already be caused by the randomness of presynaptic APs as expectable from ion-channel stochasticity (Figure 4). In such extracellular registrations (Dewald et al., 1999), it occasionally happens that APs of two or more different nerve cells can be detected. In the example of Figure 4, the activity of two different nerve cells can clearly be distinguished just by the size of the APs. The small APs appear in impulse groups, so-called “bursts,” which, as can be assumed just from the temporal sequence, trigger the single large APs of a second nerve cell, suggesting that the neuron with the small APs is synaptically innervating the neuron with the large APs.

However, the activity of the small cell is by no means completely regular. The distances between bursts vary, as do the distances of the APs within the burst – and even the number of APs per burst varies (Figure 4). Sometimes there are four and sometimes there are three impulses in a group (Figure 4, upper trace). Occasionally, there is only a short burst of only two impulses (Figure 4, lowest trace). In this case, the large AP is missing. Two successive APs seem not to lead to sufficient transmitter accumulation for sufficient postsynaptic depolarization to trigger an AP. In this case, it is clear that the uncertainty of synaptic transmission, spike or not, is not due to the synapse. Coincidence comes into play beforehand, namely, already in the triggering of more or less impulses per burst in the presynaptic cell, eventually due to the stochasticity of ion-channel states.

Of course, the presynaptic “bursting” cell is itself part of the network and is therefore also subject to the influence of other cells. It will therefore be almost impossible to determine what gives rise to the variability. However, such variability in the number of spikes per burst is also found in isolated neurons and sensory nerve endings without any synaptic contacts (Braun et al., 1980; Schafer et al., 1986) and, accordingly, could be completely sufficient to lead to uncertainties and coincidences in the synaptic transmission. This does not mean that there are no other random processes than from ion-channel noise involved in synaptic transmission as, for example, in transmitter release. At this level, they are simply no longer unambiguously detectable – in contrast to the random processes of the ion channel level as shown above.

### Neural Networks and (De)Synchronization

Simultaneous registration of APs of two different neurons in the CNS exhibiting such clear time relations as in Figure 4 is a matter of sheer luck, i.e., pure coincidence. Usually, each nerve cell, even in brain slices, is connected to so many other nerve cells that it does not matter if one of the nerve cells sends one AP more or less. In larger neuron populations, it will be almost impossible to estimate whether the observed variability reflects principle randomness or whether it is simply due to the unmanageability of such a large, multiple meshed system. In field potentials or in EEG recordings, the activity of millions to billions of neurons and synapses is comprised.

From such signals, it is mostly only possible to determine, by means of statistical methods, whether the neuronal activity is synchronized in some way, how strong the synchronization is, and what frequency range it covers. A very important and frequently addressed question is what brings neuronal population in or out of a synchronized state, e.g., during sleep–wake cycles or under pathological conditions as in Parkinson disease or epilepsy (Pikovsky et al., 2001; Uhlhaas et al., 2009).

Transitions between unsynchronized and synchronized states, for example, naturally happen every day in course of the sleep–wake cycle what can also be mathematically be simulated (Postnova et al., 2009). In computer simulations, there are several ways to bring a neural network in a synchronized state. Mostly, the synaptic connectivity is increased (Pikovsky et al., 2001; Tchaptchet, 2018), but also the transition from single spike activity to burst discharges can lead to synchronization (Postnova et al., 2007, 2009; Holmgren Hopkins et al., 2018). However, when a network once has been synchronized by certain parameter changes, it will not automatically find its way out of synchronization just when returning to the original parameter values of the previously unsynchronized state. It needs a disturbance – or noise. Deterministic systems, without an external disturbance, will stay in a synchronized state whenever they have reached such a state. By contrast, stochastic system, using noise as a disturbance, can smoothly go in and out of synchronization.

This is a functionally very important effect of randomness. Neuronal synchronization should never be complete and only temporary. Permanent synchronization, even in parts of the brain, is pathological, as is the case with Parkinson disease or epilepsy, for example. If this cannot be prevented by medication, it is often attempted to interrupt synchronization by means of direct electrical stimuli (deep brain stimulation). An external stimulus is also often used as a wake-up stimulus to get back from the partially synchronized state of sleep to the desynchronized state of wakefulness. But even without alarm clocks or other external influences, you will wake up from a restful sleep after some time.

Noise-induced phase transitions (Arhem et al., 2005), including transitions from non-synchronized to synchronized activity, may play a functionally most relevant role, not only for sleep–wake cycles but possibly even more for the “binding” of information from different sources (Singer and Gray, 1995; Engel and Singer, 2001), allowing neurons to go in and out of synchronization and to synchronize with varying partners, depending on the matching of different stimulus inputs. Stochasticity keeps the brain in a flexible state and increases its, in any case, enormous degrees of freedom practically to infinity – as far as humanly imaginable.

### Main Characteristics: Non-linear Functions – Tuned Into Noise

**FIGURE 5:**
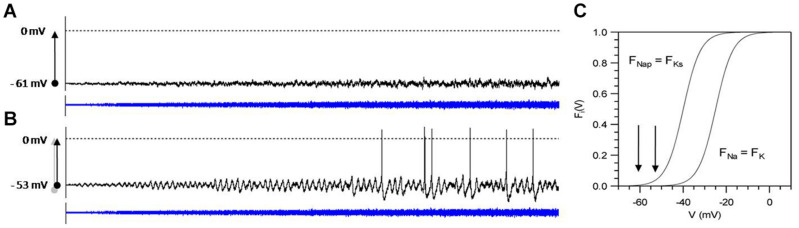
Computer simulation of noise effects on a model neuron of subthreshold oscillations and spike generation, tuned to different steady-state potentials (**A:** –61 mV, **B:** –53 mV) and subjected to noise of increasing intensity *D* (*D* = 0 to 0.4, blue curves) Simulation time: 10 s. Arrows in **(C)** indicate the position of the steady-state potentials in comparison to the activation curves *F*(*V*) of the subthreshold currents (*F*_*Nap*_, *F*_*Ks*_) and spike generating currents (*F*_*Na*_, *F*_*K*_). Model equations and parameter values are given in Huber and Braun (2006).

Biological systems are built upon multiple meshed non-linear negative and positive feedback loops. The combination of such non-linear functions can easily lead to bifurcations at which the system may switch in the one or the other direction, depending on the tiniest changes. Biological systems apparently are preferably operating in the neighborhood of bifurcations or thresholds. Ion channels, of course, have to operate close to the opening and closing threshold so that small random effects from thermodynamics can lead to switching. The thereby introduced ion-channel noise, again, can only be of relevance for AP generation if the neuron operates close to the threshold of spike generation. This is illustrated in Figure 5 with computer simulations of a Hodgkin–Huxley-type neuron for spike generation with subthreshold oscillations (Huber and Braun, 2006). In these simulations, noise of linearly increasing intensity has been added to the membrane equation. In the upper graph, this leads to nothing more than noisy fluctuations in the membrane potential. By contrast, in the lower simulation subthreshold, membrane potential oscillations become visible at very low noise intensities, which, with further increasing noise, occasionally generate APs. The only difference is that the membrane potential in the lower simulation was shifted by a constant external current further into the range of the non-linear activation curves F(V) indicated by the arrows in C.

Although both simulations start with a steady-state potential at zero noise, the model neuron in (A) does not show any functionally relevant effect, whereas in the slightly more depolarized state (B), the addition of noise leads to activity patterns of exceptional sensitivity as described for shark electroreceptors (Braun et al., 1994). These receptors, by so far unknown mechanisms, always come back into this sensitive state even after strong disturbances. Biology, in general, seems to preferably adjust their system dynamics into such noise-sensitive states. By the way, when further depolarization leads to regular spontaneous spiking already without noise, noise effect will again diminish (Jin et al., 2011).

This strategy is somehow different from stochastic resonance (SR) as the most often mentioned phenomenon in context with noise. This concept goes back to observations in the physical world but is also applicable to biological and technical systems (Douglass et al., 1993; Moss and Pei, 1995; Wiesenfeld and Moss, 1995; Gammaitoni et al., 1998; Hänggi, 2002). SR means, in short, that for certain systems there exists an optimal level of noise at which they exhibit highest sensitivity to otherwise subthreshold signals. Noise can help to bring the subthreshold signal across the system’s detection, whereby the probability of threshold crossings changes with the distance between the signal and the detection threshold. In this way, noise allows to reconstruct the form of the signal at least approximately. This works best when noise is not too low for sufficient threshold crossings and also not too high to induce too many threshold crossings independently of the signal. Hence, there is an optimal noise level somewhere in between, which leads to a typical resonance curve.

By contrast, in biology, noise is not the relevant control parameter. Noise is simply present. What has to be adjusted is the system’s operating point. The strategy in biology, partly also used for the improvement of technical sensors (Bulsara and Gammaitoni, 1996), is not “tuning noise” but “tuning INTO noise.” Indeed, you may behaviorally search for optimal randomness in your social environment, e.g., to improve your mood (Huber et al., 2000a). However, it will be difficult to adjust noise in and around your neurons. In behavioral studies, one can also try to isolate a person from all environmental influences taking him/her into a sensory deprivation chamber – with, speaking from own experience, not very pleasant effects. Environmental noise may thereby be eliminated but not the internal noise in the brain. Complete elimination of noise is possible only in mathematical models for the examination of noise effects in direct comparison with fully deterministic situations.

## Discussion

The everyday experience of free decision-making can be considered as an illusion based on the argument that, like everything in the universe, volition is totally determined by physical laws. Originally propounded by ancient philosophers, this notion received recent neuroscientific support in the form of recordings of readiness potentials preceding volitional decisions. That brain functions are fully deterministic is challenged by experimental neurophysiological data, supplemented by computer simulation, which demonstrate the physiologically relevant contribution of randomness at all levels of brain function. Special emphasis has been laid on the well-known occurrence of randomness at the lowest level of neuronal information processing, the opening and closing of ion channels, and that this randomness or noise is not necessarily smeared out at higher levels but can even be enhanced by appropriate adjustment of the system’s non-linearities.

The need of a disturbance for network desynchronization, at least in form of noise, is only one example of the functional implications of neural stochasticity at higher organizational levels, such as controlling sleep–wake cycles. Usually, such disturbances come from outside the body (Freeman, 2000; Buzsaki, 2006; Isomura et al., 2006), but externally evoked disturbances have been excluded in this study. The aim of these studies was to focus on the inherent stochasticity of neural functions and how processes, even at this level, may allow escape from a fully deterministic physical world.

Most studies in search of evidence to counter the notion of fully deterministic brain functions have so far investigated stochasticity in synaptic transmission or irregularities in neuronal firing times (Heisenberg, 2009; Rolls, 2012). However, this approach is confounded by the complexity of even a single neuron or synapse such that any observed unpredictability may simply be due to our lack of knowledge of the “uncontrollable perturbations originating from the system’s environment or intrinsic interdependencies among its internal constitutions” (Atmannspacher and Rotter, 2008). Moreover, considering the enormous number of neurons and their multiple, random-like fluctuations in compound potentials as in the EEG can be expected even when all functions may be fully deterministic.

### The Need of Appropriately Adjusted System Dynamics

These studies began with the lowest level of neural function, the opening and closing of voltage-gated ion channels. Even at this level, there is substantial complexity, such as the control by gating currents that, in turn, operate in a principally uncontrollable environment even in perfectly designed experiments. At the level of ion channels, however, stochasticity is the mechanistically determinant factor. Ion-channel opening and closing are not superposed by randomness. They are random!

At the next higher level, the generation of APs, randomness is a less determinant factor. It is not so much influencing the shape of an AP but can decide about AP generation. Although AP generation still fully depends on the opening and closing of individual ion channels, it is the compound effect of ion channels that becomes the relevant factor and then manifested in random fluctuations, i.e., noise (Figures 3, 5). Depending on the individual neuron’s dynamics, this leads to more or less irregular spiking patterns.

In neuronal networks, ion-channel stochasticity may appear in a different form, although no longer distinguishable from otherwise introduced irregularities. Already the manifold of synaptic connections can lead to network oscillations and to the appearance of a particular kind of “noise” (Buzsaki, 2006, 2007). Ion-channel stochasticity can directly come in through the diversity of ion channels in the presynaptic and postsynaptic membranes and can indirectly contribute via the irregularities of individual neurons’ firing patterns.

Many neurons in the brain, because of their more complex dynamics, generate a much greater variety of firing patterns than shown in figures of this study with an accordingly greater variety of noise effects (Braun et al., 2003). These can become particularly pronounced at the transitions from single spike activity to burst discharges (Finke et al., 2008), as in critical functional transitions as such as the occurrence of epileptic seizures or in the course of sleep–wake cycles (Postnova et al., 2011). Thus, as the stochasticity of ion channels propagates through higher functional levels, from the microscopic to mesoscopic to macroscopic levels, it becomes manifest in different forms and combinations that are additionally modulated by manifold networks of feedforward and feedback loops.

It is important to note that non-linearities are not a sufficient prerequisite for effective propagation of noise. The important point is that that the systems operate in the neighborhood of bifurcations. This has been demonstrated by the simulations in Figure 5 showing negligible effects of noise in one simulation, whereas the second simulation of the same model neuron subjected to the same noise but with a slightly shifted operating point develops spiking in a functionally relevant pattern as observed in many peripheral and central neurons (Alonso and Klink, 1993; Klink and Alonso, 1993; Braun et al., 1994; Huber et al., 2000c). Many neurons exhibit broad ranges of more or less regular spiking and bursting activity with only minor but recognizable signs of randomness. Such rather regular, periodic pattern can alternate with rather unpredictable spike pattern (Braun et al., 2003; Mosekilde et al., 2004). The impact of stochasticity clearly depends on the neuron’s dynamic state, which is determined by physiological control parameters.

The same rules hold true for ion channels, probably with greater, fundamental impact. If ion channels are not operating close to the switching threshold, molecular fluctuations will not influence opening or closing of the channel. There are many ion channels that are closed and are activated only under specific conditions, such as at specific membrane potentials or in response to specific neurotransmitters and neuromodulators. But, once activated, their switching depends on stochastic processes. As shown in Figure 1, ion-channel transitions are highly temperature-sensitive, a characteristic that is clearly consistent with switching being determined by Brownian motion.

These findings suggest that biology, specifically neurophysiology, harnesses stochasticity in a particular way, i.e., adjusting their system dynamics into noise-sensitive states. This begins with an appropriate adjustment of ion channels. This leads to non-linear, approximately sigmoidal activation curves of ion currents superposed by the simultaneously introduced noise. The neurons can then be tuned to a state in which AP generation is subjected to more or less pronounced stochasticity. The stochasticity of the neuron’s firing pattern directly contributes to the stochasticity of synaptic transmission together with the inherent randomness of diverse ion channels at the transmission sites. Stochasticity can be further enhanced in neuronal networks by the manifold of synaptic feedforward and feedback connections. This maintains the brain in a flexible state and allows switching between multiple, coexisting decision options in the control of autonomic, mental, and also cognitive functions.

This does not mean that the neurons’ intrinsic randomness is always a decisive factor for all neurons for spike generation and synaptic transmission. At a given time, a significant portion of the neurons may be silent, with membrane potentials far below the spiking threshold, whereas others may operate clearly above the threshold generating regular spike patterns. These are both comparably stable states. However, there are still a significant number of neurons operating in the intermediate range in which stochastic effects can determine about spiking and the spiking pattern, thereby introducing randomness in connected neurons, which likewise operate in the neighborhood of bifurcations (Figure 3). These are the dynamic states in which the neurons are highly sensitive not only to noise but also to all kinds of signal inputs.

The dynamics of individual neurons are not fixed, but can shift between comparably stable and highly sensitive states under the influence of neurotransmitters and neuromodulators. It is completely unclear in which way specific neurons can be tuned to a state appropriate for a specific task or whether stochasticity can be specifically enhanced for particularly difficult decision-making. The system may reach a conclusive state just by floating around. At which points and under which conditions this leads to a decision and an action by the agent are far from being understood.

Indications of intrinsic adaptive processes contributing to an appropriate adjustment could so far only be observed at the single neuron level, e.g., in recordings from shark electroreceptors (Braun et al., 1994). These receptors are always adapting, even after very strong stimuli, to their most sensitive state of spike generation with subthreshold oscillations and noise. Adaptation, indeed, is a ubiquitous feature of neuronal transduction, specifically well-known from sensory receptors. However, it is completely unknown which mechanisms may be behind such a seemingly target-oriented adaptation process.

Altogether, the here presented concept and data lead to many additional questions, which so far cannot be answered. Many of these questions have probably not yet been asked. There are many studies of possible noise effects in the brain mostly with regard to specific functions (White et al., 1998; Huber et al., 2000b; Zhou and Kurths, 2003; McDonnell and Ward, 2011; Fisch et al., 2012). However, the concept presented here that the intrinsic stochasticity of neural function contributes to keep the brain in a flexible state also for deliberated decision-making appears not yet to have been seriously considered.

### Stochasticity, Readiness Potentials, and the Free Will

Demonstrating a functionally relevant role of principally unavoidable stochasticity in neuronal information processing cannot prove the existence of free will. However, it is similarly questionable whether recordings of EEG or fMRI signals (Libet, 1982; Libet et al., 1982, 1983a,b; Soon et al., 2008; Schultze-Kraft et al., 2016) are really a conclusive means of determining the presence or lack of free will (Mele, 2014).

Of course, it must be expected that any volitional action such as moving a finger should be preceded by brain activity followed by APs traveling along the peripheral nerves to activate the required muscles. Such “readiness potentials” in the brain were recorded and described by Kornhuber and Deecke (1965). What was new in the subsequent findings of Libet et al. (1982, 1983a) was that the test subjects were asked to watch a clock and tell the experimenter the precise time at which they decided to press the button. Surprisingly, this was several hundred milliseconds later than the EEG signals have been recorded. Delays of up to several seconds have been observed in BOLD signals in more recent fMRI studies of the Haynes group (Soon et al., 2008), what appears even more surprising as the BOLD signal itself lags several seconds behind the hemodynamic changes in the brain it measures.

These studies have been criticized from different points of view not only concerning methodological issues but also an overinterpretation of such data (Mele, 2014). A major question is to what extent such signals of unconscious brain activity can be related to conscious decisions (Nussbaum and Ibrahim, 2012), what leads to particularly difficult issues such as the “hard problem” of consciousness or, more general, the mind–body problem (Clement et al., 1998; Freeman, 2000; Blackmore, 2005; Buzsaki, 2007; Mele, 2014; Kotchoubey et al., 2016; Liljenstrom, 2020), which are beyond the scope of this study.

Several more recent recordings of brain signals are coming closer to the questions of unconscious versus volitional actions comparing brain signals advancing spontaneous activity and self-initiated movements (Schurger et al., 2012; Uithol and Schurger, 2016; Travers et al., 2019). Other studies suggest “different neural mechanisms underlying deliberate and arbitrary decisions” also bringing noisy, random fluctuation into the play (Maoz et al., 2019a,b). It is suggested that random fluctuations drive arbitrary but not deliberate decisions. The reason could be that the intrinsic stochasticity of brain dynamics may become more recognizable in arbitrary compared to deliberate decisions. Decisions are never purely random but may take advantage of neural stochasticity to explore varying brain states in goal-directed searches for appropriate actions. According to Walter Freeman and others, the brain is not primarily an information-processing system but more an information-searching or, even more, a meaning-searching system (Freeman, 2000).

It seems that different approaches, such as recordings of brain signals and examinations of basic neuronal processes, come closer together complementing each other, also concerning the question of a free will. It could be advantageous to lay more emphasis on multilevel feedback mechanisms connecting organic and mental functions with cellular and molecular processes, bottom-up as well as top-down (Freeman, 2000; Arhem et al., 2005; Noble, 2006; Murphy et al., 2009; Braun, 2015; Liljenstrom, 2018; Noble and Hunter, 2020).

### Stochasticity in Biology Compared to the Physical World and Engineering

Biology has apparently learned in the course of evolution to harness stochasticity by tuning its functions into a noise-sensitive range. Such functionally relevant interactions between physiological laws and stochasticity are not limited to neurobiology. In 1971, Jacques Monod published his spectacular book “Chance and Necessity” (Monod, 1971), emphasizing the impact of chance in evolution, although not so much highlighting a possibly advantageous role. The “Blind Watchmaker” by Richard Dawkins (Dawkins, 1971) goes in a similar, leaving almost everything to randomness – under subsequent control by natural selection.

The concept of harnessing stochasticity in evolution, taking advantage of the interplay of coincidence and lawfulness under environmental pressure, has more recently been proposed by Raymond and Denis Noble (Noble, 2017; Noble and Noble, 2017, 2018). They also present several examples of how organisms can make choices out of random variations for the control of autonomic functions under the pressure of physiological demands, e.g., to achieve appropriate immune responses.

Altogether, there can be no doubt that stochasticity plays a prominent and functionally relevant role at all levels and processes of living systems, from evolution to neural information processing, and presumably including conscious decision-making. One may ask whether this is due to general randomness in the universe and the fundamental unpredictability of physical laws.

This report concerns randomness at the molecular level and specifically Brownian motion. If it is said “Our world is either deterministic or indeterministic, but not both” (Muller et al., 2019), this may be formally correct. However, even when determinacy in the physical world could undoubtedly be proven, including the movements of each single molecule, certain unpredictability remains in their compound effects, manifested as random fluctuations, which is apparently sufficient for biology to take advantage of it. Laplace demon is metaphysics (Russell, 1959). What greater degrees of freedom could exist than only limited by the knowledge about the precise position and momentum of all particles in the universe?

Brownian motion is omnipresent, also in the physical world, which means that there is no need assuming biology-specific randomness to account for the particular stochasticity in neuronal systems. If coincidence nevertheless seems to play an even greater role in living creatures than in inanimate nature, this could, of course, be due to the fact that the animate world is much more complex and much less transparent than the physical world. However, as the most important point, the here presented data suggest that it is rather the functional organization of biological systems tuning the systems’ dynamics into noise-sensitive states, what makes them particularly susceptible to stochasticity, apparently with certain advantages.

For electrical engineers, it is among the main topics of their education to learn, apart from specific tasks, how to suppress noise and how to linearize to avoid unpredictable situations including undesired oscillations and chaotic dynamics. By contrast, biology is full of noise, non-linearities, oscillations, and chaos. Moreover, information processing happens on a much slower time scale compared to light velocity of electromagnetic signals in technical artifacts. Nevertheless, there is not any technical system so far, including recent developments in artificial intelligence, which could compete with the flexibility and creativity of the human brain. Maybe addition of noise with appropriately adjusted non-linearities could help. However, who would like, even when looking forward to self-driving cars, that these also develop their own free will? It is often complicated enough to deal with all the people around who all have their own (free?) will.

### General

The physiological correlates of free will, the capacity to make conscious volitional decisions, are still unknown. It remains unclear how human agents make these decisions. The classic mind–brain problem and its implications for free will are far away from a solution.

None of these problems can be solved either by recordings of readiness potentials or by demonstrating the propagation of ion-channel stochasticity. It is even questionable whether any experimental recordings can help to decide whether human decisions and volitional actions are free or not.

Nevertheless, it is still an important task in neuroscience to achieve a more thorough understanding how human beings make their decisions and what brain mechanisms thereby play a major role, also with regard to mental disturbances. This may approximate an answer to the question of whether human agents have free will.

This definitely needs to look at different levels of brain function at the microscopic, mesoscopic, and macroscopic scale. Such an attempt was made with the presented study here, however, with a very limited focus only on the propagation of ion-channel stochasticity. Other studies are mainly referring to unconscious signals in readiness potentials preceding quite simple volitional actions. Many studies address such questions from a rather philosophical point of view up to the hard problem of consciousness and the mind–brain problem.

Hence, for better progress, it will probably be necessary not only to connect the different scales but also the different methods, repeatedly proposed but hardly realized. Bringing together the different groups from different fields for combined efforts is not an easy task. The different groups seem to speak in different languages and do not easily understand each other. Nevertheless, it still may be worth making further efforts to overcome these “Methodological Problems on the Way to Integrative Human Neuroscience” (Kotchoubey et al., 2016).

## Data Availability Statement

Simulation data can be delivered as well as the equations and parameter values. Raw data of the experiments from more than 20 years ago, also basis of previous publications, are on analog tape, probably difficult to read nowadays (see Ethics Statement).

## Ethics Statement

All our experiments to which this article refers have been done more than 20 years ago (Braun et al., 1994; Dewald et al., 1999).

## Author Contributions

The author confirms being the sole contributor of this work and has approved it for publication.

## Conflict of Interest

The author is co-owner of BM&T and main developer of the Virtual Physiology labs with benefits from the author’s experiences in education and research.
